# Evidence for multiple-insecticide resistance in urban *Aedes albopictus* populations in southern China

**DOI:** 10.1186/s13071-017-2581-y

**Published:** 2018-01-03

**Authors:** Yiji Li, Jiabao Xu, Daibin Zhong, Hong Zhang, Wenqiang Yang, Guofa Zhou, Xinghua Su, Yang Wu, Kun Wu, Songwu Cai, Guiyun Yan, Xiao-Guang Chen

**Affiliations:** 1 Department of Pathogen Biology, Guangdong Provincial Key Laboratory of Tropical Disease Research, School of Public Health, Southern Medical University, Guangzhou, China; 2 Key Laboratory of Translation Medicine Tropical Diseases of Ministry of Education, Hainan Medical University, Haikou, Hainan China; 3 Department of Pathogen Biology, Hainan Medical University, Haikou, Hainan China; 4 Program in Public Health, School of Medicine, University of California, Irvine, USA; 5 Department of Vector Control, Centers for Disease Control and Prevention of Guangdong Province, Guangzhou, China

**Keywords:** *Aedes albopictus*, Insecticide resistance, Biochemical assay, *Kdr*, Urbanization, Guangzhou

## Abstract

**Background:**

*Aedes albopictus* (Skuse) is an invasive mosquito that has become an important vector of chikungunya, dengue and Zika viruses. In the absence of specific antiviral therapy or a vaccine, vector management is the sole method available for reducing *Aedes*-induced disease morbidity. Determining the resistance status of *Ae. albopictus* to insecticides and exploring the resistance mechanisms is essential for future vector control planning.

**Methods:**

*Aedes albopictus* larvae and pupae were sampled from six sites (two sites each from urban, suburban and rural) in Guangzhou. The resistance bioassays were conducted against *Bacillus thuringiensis israelensis* (*Bti*): deltamethrin, propoxur and malathion for larvae; and deltamethrin, DDT, propoxur and malathion for adults. P450 monooxygenase (P450s), glutathione S-transferase (GSTs) and carboxylesterase (COEs) activities of adult mosquitoes were measured. Mutations at the knockdown resistance (*kdr*) gene were analyzed, and the association between *kdr* mutations and phenotypic resistance was tested.

**Results:**

Adult bioassays revealed varied susceptibility against DDT, deltamethrin and propoxur in the six *Ae. albopictus* populations. Significantly lower mortality rates were found in urban populations than suburban and rural populations. Urban mosquito populations showed resistance against DDT, deltamethrin and propoxur, while one rural population was resistant to DDT. All populations tested were susceptible to malathion. Larval bioassays results indicated that all populations of *Ae. albopictus* were sensitive to the larvicide *Bti* and malathion. Resistance to deltamethrin and propoxur was common in larval populations. The F1534S and F1534 L mutations were found to be significantly associated with deltamethrin resistance. Biochemical assays indicated elevated detoxification enzyme activities in the field mosquito populations.

**Conclusions:**

*Aedes albopictus* populations in Guangzhou, especially in urban areas, have developed resistance to the commonly used insecticides, primarily DDT and deltamethrin. This finding calls for resistance management and developing counter measures to mitigate the spread of resistance.

**Electronic supplementary material:**

The online version of this article (10.1186/s13071-017-2581-y) contains supplementary material, which is available to authorized users.

## Background

*Aedes albopictus* (Skuse) (Diptera: Culicidae), the Asian tiger mosquito, is an important vector of dengue, chikungunya, yellow fever and Zika viruses, which have emerged as global public health threats [1–5]. This mosquito originated at the edges of forests and bred in natural habitats, and it was previously considered a rural vector [6]. However, *Ae. albopictus* has adapted well to urban environments with larvae now breeding in artificial containers and has become the most important and sometimes sole vector in urban areas [7, 8]. *Aedes albopictus* is the primary dengue vector in China [4, 9, 10]. Guangdong Province experienced the highest incidence of dengue in mainland China in the past 40 years [11] accounting for 90% of dengue cases in China. Several major dengue fever outbreaks have occurred in this area since 1978, and *Ae. albopictus* was the sole vector. Since the 1990s, more than 30,000 dengue cases were reported in Guangzhou. Most of the dengue cases are present in urban areas of Guangzhou [12].

In the absence of specific antiviral therapy or a vaccine, control of *Ae. albopictus-*borne diseases by vector management is the sole method available for reducing the disease burden. Adult mosquito control depends largely on insecticides. However, resistance to insecticides is rising globally [13–18]. The extensive use or non-regulated application of pesticides can hamper the efficacy of larvicide and adulticide-based control programs, as demonstrated in the vector control of *Ae. aegypti* [19, 20] and *Culex pipiens quinquefasciatus* [21]. It is reported that *Ae. albopictus* is resistant or incipient to the major insecticides currently or historically used for vector control across the world, i.e. DDT [16, 20, 22–25], malathion and bendiocarb [23] and pyrethroids [26, 27] such as permethrin [14, 22, 23] and deltamethrin [16, 22, 23]. Previous studies indicated that *Ae. albopictus* in Guangzhou was sensitive to all types of insecticides prior to 2002 [28]. Along with the rapid urbanization and recent regional economic development, insecticides were extensively and frequently used in Guangzhou city for dengue control in the past decade [29]. Recent studies have demonstrated that *Ae. albopictus* developed resistance against pyrethroids during the period when pyrethroids had been massively applied in Guangzhou [29–31].

Global surveys indicated that insecticide resistance in mosquitoes can be associated with target-site insensitivity, and/or metabolic-based detoxification. The main target site inactivity mechanisms involve (i) amino acid substitutions in the voltage gated sodium channel (*VGSC*) that cause a resistance phenotype to pyrethroids and DDT insecticides known as knockdown resistance (*kdr*) [32]; and (ii) mutations in the acetylcholine esterase sequence that lead to insensitivity of this enzyme to organophosphates [33]. Metabolic detoxification has been found to be a key resistance mechanism in *Anopheles* and *Culex* mosquitoes [34, 35]. Detoxification enzymes typically linked to insecticide resistance mainly include three major gene families, cytochrome P450 monooxygenases (P450s), carboxylesterases (COEs), and glutathione S-transferases (GSTs). So far, compared to other mosquito species of public health importance such as *Anopheles* spp., *Culex* spp. and *Ae. aegypt*i, very little is known about the molecular or biochemical basis of resistance in *Ae. albopictus*. Previous studies have examined general resistance status in limited number of mosquito populations in Guangzhou city. A systematic examination of *Ae. albopictus* resistance status and mechanism investigation in different ecological settings would provide important information on resistance distribution and guidance on resistance management.

In this study we explored insecticide resistance of larval and adult *Ae. albopictus* in different settings (urban, suburban and rural) in Guangzhou. We adopted biochemical and molecular assays to identify putative resistance mechanisms in *Ae. albopictus* adult for target-site mutations and detoxifying enzymes up-regulation. We also investigated the insecticide application and sales in different settings in Guangzhou.

## Methods

### Study sites

**Fig. 1:**
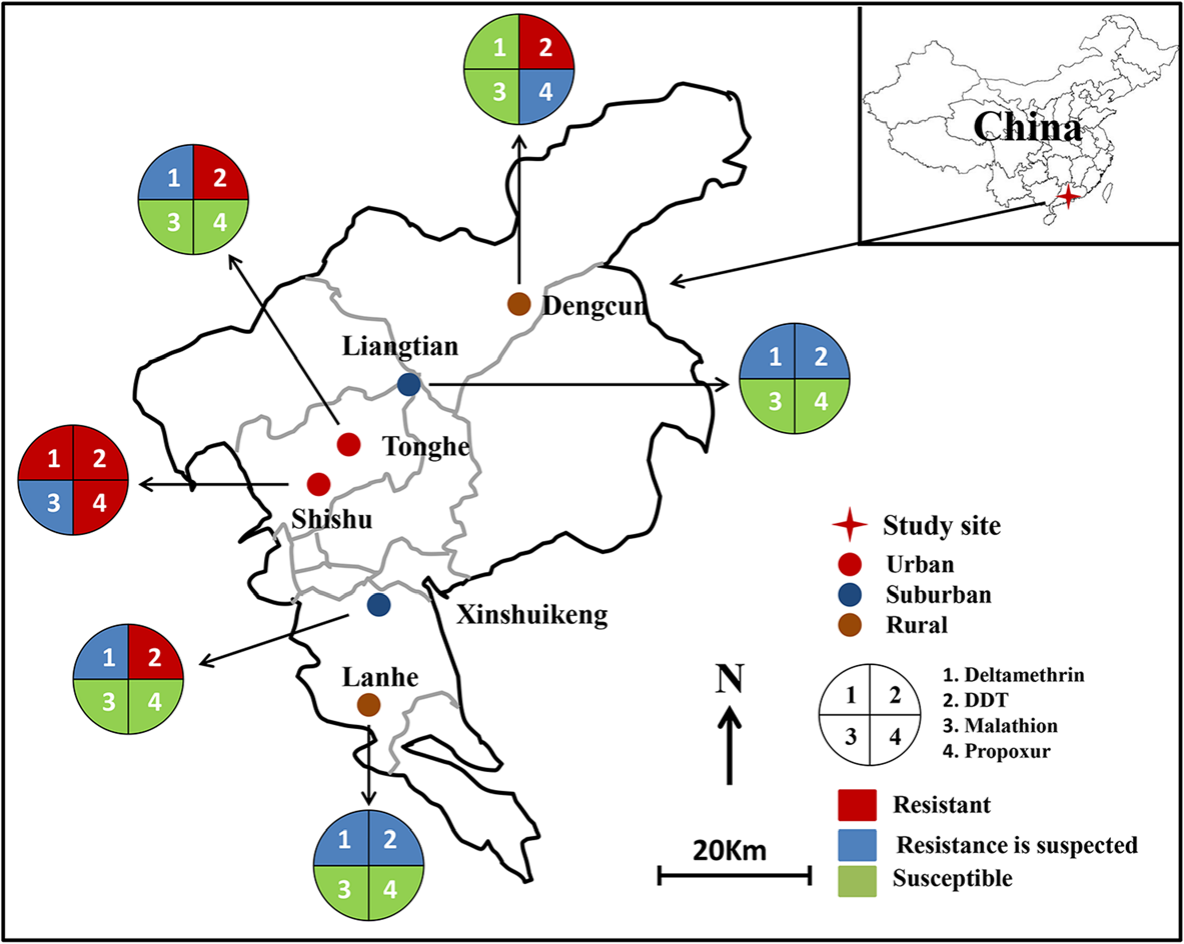
Map of study areas and insecticide resistance status of adult *Aedes albopictus* in three ecological settings in Guangzhou, China

The study was conducted in dengue endemic areas in Guangzhou, Guangdong Province, China (Table 1, Fig. 1). Guangzhou, about 200 km north of Hong Kong, is the largest city in Guangdong Province and key commercial harbor in southern China, with a population of 12 million according to the 2012 census survey [36]. It is located in the sub-tropical area with an annual average temperature of 21.6 °C, and annual cumulative precipitation of about 1980 mm. We selected six sites, two each in urban, suburban and rural areas, for our study.

**Table 1:** Description of *Aedes albopictus* mosquito population collection sites from urban, suburban and rural settings in Guangzhou, China

Geographical classification	District	Village	Mosquito population ID	Coordinates		Altitude (m)	Human density (inhabitants/km^2^)
				Latitude (°N)	Longitude (°E)		
Urban	Baiyun	Tonghe	UBT	23.18522897	113.328463	40	> 3000
	Yuexiu	Shishu town	UYS	23.12603999	113.251287	32	> 3000
Suburban	Baiyun	Liangtian	SBL	23.35942101	113.368739	38	*c.*1000
	Panyu	Xinshuikeng	SPX	22.96891904	113.390611	20	*c.*1000
Rural	Conghua	Dengcun	RCD	23.49894198	113.553073	27	< 300
	Panyu	Langhe	RPL	22.82577298	113.342364	31	< 300

### Mosquito strains and collection

Six populations of *Ae. albopictus* larvae were collected from different ecological settings, i.e. two each in urban, suburban, and rural, from May to October 2014 (Table 1, Fig 1). Tonghe town in Baiyun district (23°11′24″N, 113°19′48″E, 40 m above sea level, masl) and Shishu town in Yuexiu district (23°07′48″N, 113°15′0″E, 32 masl) are urban areas with a population density of > 3000 people/km^2^. Land usage types are primarily residential and commercial buildings as well as public services such as schools and hospitals, filled with trees and grasses. Liangtian town in Baiyun district (23°21′36″N, 113°22′12″E, 38 masl) and Xinshuikeng town in Panyu County (22°58′12″N, 113°23′24″E, 20 masl) are suburban areas with a population density of approximately 1000 people/km^2^, and land use includes a mixture of residential, manufacturing, and farmland. Dengcun village in Conghua County (23°30′0″N, 113°33′0″E, 27 masl) and Lanhe village in Panyu County (22°49′48″N, 113°20′24″E, 31 masl) are rural areas with a population density of < 300 people/km^2^, where land is primarily used for agriculture such as rice and vegetable farming, and forest.

### Insecticide resistance bioassays

#### Larval resistance bioassays

Four insecticides of technical grade were used. Three chemical insecticides were from the Chinese Center for Disease Control and Prevention (China CDC): propoxur (95.56% pure), deltamethrin (94.62% pure) and malathion (95% pure), and one microbial larvicide: *Bacillus thuringiensis israelensis* (*Bti*) (7000 ITU/mg, Wuhan Nature’s Favour Bioengineering Co., Ltd., Wuhan city, China). All insecticides except *Bti* (which was diluted in water) were diluted in acetone to the required dosage following WHO guidelines [37]. All bioassays were performed using *Ae. albopictus* collected directly from the field after species identification. A set of 25 third- and fourth-instars larvae was incubated in 99 ml of distilled water, to which 1 ml of insecticide solution at the required concentration was added. Three replicates were tested for each concentration. Five to nine concentrations, providing a range of mortalities between 10 and 95%, were used to determine LC_50_ values (the 50% mortality lethal concentration). Mosquitoes of the Foshan strain, which have been reared in the laboratory since 1981 without insecticide exposure, were used as a control. Larval mortality was recorded after 24 h exposure. Control bioassays were conducted by adding 1 ml of acetone to 99 ml of distilled water. Temperature and relative humidity were maintained at 27 ± 2 °C and 80–90%, respectively, in an incubator with a 16:8 h light:dark period.

#### Adult resistance bioassays

For each strain, five batches of 20 non-blood-fed females (3–5 day-old; *n* = 100) reared from field-collected immature *Ae. albopictus* were subjected to insecticide susceptibility test against 0.05% deltamethrin, malathion (0.8%), propoxur (0.1%) and DDT (4%) following the standard WHO tube test protocol [38]. Briefly, we defined resistant mosquitoes as mosquitoes that survived 24 h after the end of the bioassay, and susceptible mosquitoes as the mosquitoes that were knocked down during the 60 min exposure time or that died within the 24 h recovery period. Mosquitoes were considered knocked down if they were unable to fly when they were mechanically stimulated. [39] Mosquitoes of the Foshan strain, which have been reared in the laboratory since 1981 without insecticide exposure, were used as a control (hereafter referred to as laboratory susceptible strain). WHO test and control papers were supplied by China CDC, except for deltamethrin which was supplied by the School of Biological Sciences, Universiti Sains Malaysia (11800 Minden, Penang, Malaysia). The knockdown time (KDT) of females was reported every 10 min during the 60 min exposure period. Mortality was scored after the 24 h recovery period. After the bioassay, one leg of each mosquito was removed and stored individually in 95% alcohol for subsequent DNA analysis.

#### Metabolic enzyme activity assays

Three metabolic enzymes, P450s, GSTs, and COEs, were analyzed on single individuals from 3 to 5 day-old F0 females without insecticide exposure, and on the laboratory susceptible strain following previously published protocols [40]. Briefly, mean absorbance values for each tested mosquito and enzyme were converted into enzyme activity and standardized based on the total protein amount. Total protein was measured for each mosquito using the method of Bradford [40]. All measurements were done in duplicate. COE activity was measured following the method of Hosokawa & Satoh [41]. Spontaneous hydrolysis was used as a blank control. COE activity was calculated as μmol of p-nitrophenol formed per min per mg protein, using the formula: Δabsorbance/min – Δblank/min) × 1.0/16.4 × 0.05 × protein (mg/ml). An absorption coefficient of 16,400 M was used [42]. For each mosquito population and each insecticide, 30 female adult mosquitoes were tested.

### DNA extraction and *kdr* mutations detection

Genomic DNA was extracted from individual mosquitoes using Fast Tissue-to-PCR Kit (Sigma-Aldrich, Missouri, USA) following the product protocol. Extracted DNA was stored at 4 °C or used immediately for PCR. All survivors and 40 randomly selected (when available) dead specimens after DDT and deltamethrin bioassay exposure were genotyped at the *VGSC* gene to detect mutations within domains II, III and IV, by direct sequencing of PCR products that contained the specific domains following previously published protocols [43]. Sequences were aligned and analyzed with Sequencher 5.0 (Gene Codes, Ann Harbor, Michigan, USA).

### Insecticide usage and sales survey

An insecticide sales and usage survey was conducted in May 2014 with a usage questionnaire from individual residents, the community, and agriculture, as well as insecticide sales from the shops. Insecticides usage included brand name, component content and frequency of application for agricultural and/or public health. At each site, residential insecticide usage surveys were administered to 80 households, 20 households for agriculture application, 8 communities for adult and larvae mosquito control, and 10 shops were administered insecticide sales surveys.

### Statistical analysis

Mosquito resistance status was classified based on the 2013 WHO standard [39]: resistant if mortality is less than 90%, probable resistant if mortality between 90 and 97%, and susceptible if mortality > 97%. Larval median lethal concentration (LC_50_) and adult 50% knockdown times (KDT_50_) were estimated with the log-probit model [44]. For the same insecticide, among-site difference in LC_50_ was tested by pair-wise comparison of the regression slopes of the Probit model. Intensity of resistance was measured using resistant ratio (RR_50_) defined by (LC_50_ of field population)/(LC_50_ of susceptible population) for insecticide concentration, or by (KDT_50_ of field population)/(KDT_50_ of susceptible population) for knockdown time. Generalized linear model (GLiM) was used to examine whether adult mortality in the WHO standard tube bioassay differ significantly among localities. Association between *kdr* mutations and resistance phenotype was examined using Fisher’s exact test, and odds ratio was calculated for each *kdr* allele. Statistical comparisons of detoxification enzyme levels between the laboratory susceptible strain and the field populations were assessed with the Student’s t-test. In the case that multiple comparisons were conducted, the significance level was adjusted accordingly.

## Results

### Larval resistance bioassays

Using the resistance ratio of 2.0 as the threshold value for declaring resistance for mosquito larvae, all mosquito populations were susceptible to *Bti* with RR_50_ ranging from 0.39 to 1.06, and susceptible to malathion with RR_50_ ranging from 0.74 to 1.94 (Table 2). All six populations were resistant against deltamethrin, with LC_50_ ranging 0.011 to 0.038 mg/l and RR_50_ ranging from 11 to 38 (Table 2). The urban population exhibited the highest LC_50_ in testing against deltamethrin and propoxur.

**Table 2:** Resistance bioassay results of larval *Aedes albopictus* in urban, suburban and rural settings in Guangzhou, China. Sites in the same column connected by different letters represent a significant difference in resistance levels at *P* < 0.05

Location	Population name	*Bti*		Deltamethrin		Propoxur		Malathion	
		LC_50_ (95% CI) (mg/l)	RR_50_	LC_50_ (95% CI) (mg/l)	RR_50_	LC_50_ (95% CI) (mg/l)	RR_50_	LC_50_ (95% CI) (mg/l)	RR_50_
Urban	UBT	0.016 (0.009–0.026)	0.4	0.016 (0.010–0.026)^bc^	16.0*	2.29 (1.58–3.06)^ab^	2.6*	0.177 (0.159–0.198)	1.3
	UYS	0.035 (0.030–0.040)	1.0	0.038 (0.032–0.046)^a^	38.0*	3.29 (2.47–4.39)^a^	3.7*	0.260 (0.227–0.301)	1.9
Suburban	SBL	0.038 (0.024–0.058)	1.1	0.017 (0.014–0.022)^b^	17.0*	1.52 (1.09–2.08)^c^	1.7	0.109 (0.096–0.125)	0.8
	SPX	0.016 (0.012–0.021)	0.4	0.011 (0.009–0.013)^c^	11.0*	2.14 (1.89–2.40)^b^	2.4*	0.099 (0.088–0.113)	0.7
Rural	RDS	0.014 (0.007–0.002)	0.4	0.014 (0.007–0.024)^bc^	14.0*	1.41 (1.25–1.59)^c^	1.6	0.145 (0.106–0.204)	1.1
	RPL	0.014 (0.009–0.021)	0.4	0.022 (0.020–0.026)^b^	22.0*	2.87 (2.53–3.19)^a^	3.3*	0.217 (0.171–0.282)	1.6
Lab strain	LSS	0.036 (0.028–0.047)	1	0.001 (0.001–0.001)	1	0.879 (0.802–0.952)	1	0.134 (0.121–0.149)	1

*Abbreviations*: *LC*_*50*_ lethal concentration that kills 50% of the population (mg/l), *RR*_*50*_ resistant ratio, *LC*_*50*_ field population/LC_50_ susceptible strain, *Bti* 7000 ITU/mg

*Significant resistance compared to the laboratory susceptible strain as indicated by at least a 2-fold higher RR_50_ value relative to the laboratory susceptible strain

### Adult resistance bioassays

Using the 90% mortality rate resistance threshold value designated by the WHO [45], two urban and one rural *Ae. albopictus* populations were resistant against DDT. Populations from urban areas had the lowest mortality rates against DDT (75–80%). Only one urban population showed resistance to deltamethrin, and this population was also resistant to propoxur (Additional file 1: Table S1). All six populations were susceptible to malathion. Moreover, KDT_50_ of field *Ae. albopictus* populations were longer than those of control population when exposed to DDT, deltamethrin, and malathion, as indicated by KRR_50_ > 1.0 for field populations from all sites (Additional file 1: Table S1).

### Metabolic enzyme activities and association with resistance

**Fig. 2:**
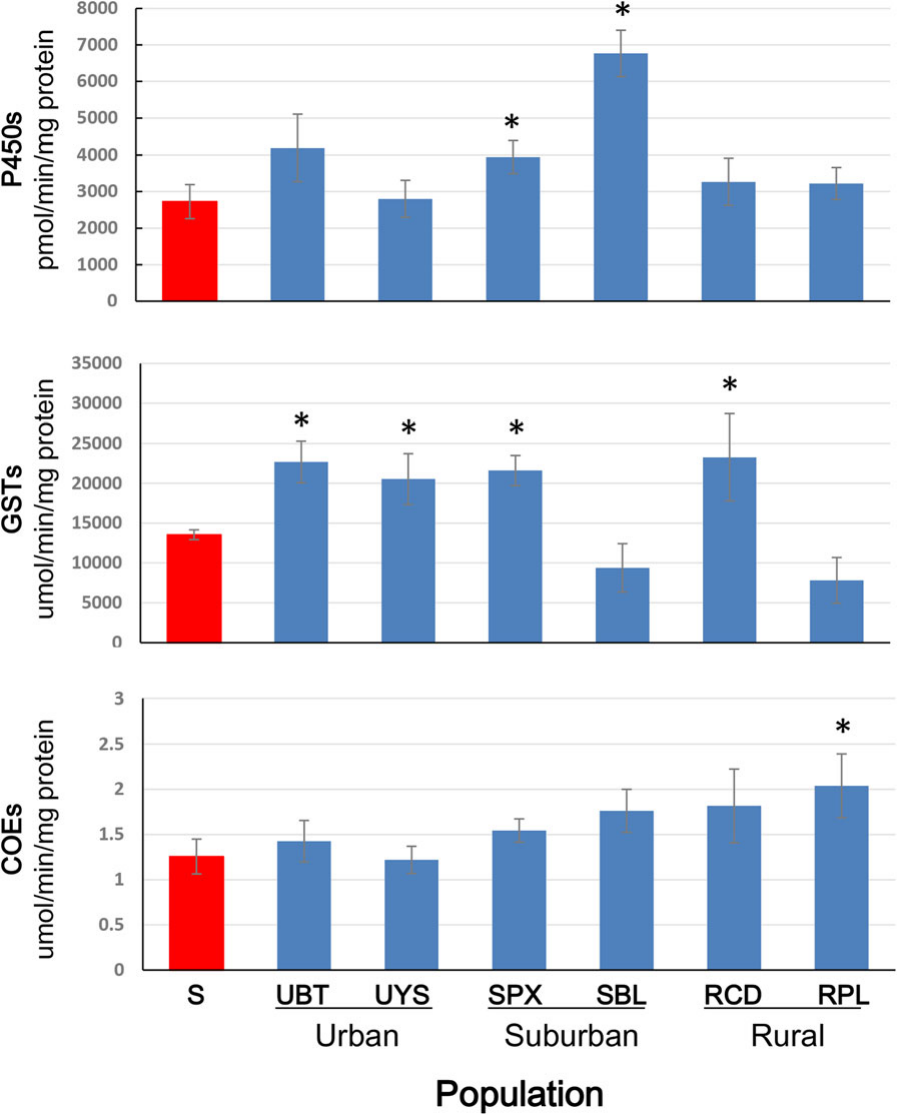
Activity of detoxification enzymes in field collected *Aedes albopictus* adult mosquitoes from Guangzhou, China in comparison to the laboratory susceptible strain. Error bar is the standard error of the mean. An asterisk (*) indicates a significant difference between the population and the laboratory susceptible strain at *P* < 0.05. *Abbreviations*: P450, monooxygenases (P450s); COEs, carboxylesterase; GSTs, glutathione-S transferases; S, laboratory susceptible strain

Comparison of the detoxification enzyme activity among the field populations and the laboratory susceptible strain without insecticide exposure found that P450 levels were significantly higher in adults from SPX and SBL (*t*_(39)_ = 1.87, *P* = 0.034; *t*_(33)_ = 5.26, *P* < 0.001) (Fig. 2). GST levels were significantly higher in four populations, and COE levels were significantly higher in one population (*t*_(39)_ = 2.11, *P* = 0.021) (Fig. 2) compared to the laboratory susceptible strain.

### *kdr* genotyping

Sequences of domains II (480 bp), III (2347 bp) and IV (280 bp) of the *VGSC* gene were obtained from a total of 111 resistant or susceptible mosquitoes after deltamethrin resistance bioassay and 305 individuals after DDT resistance bioassay. All mutations in codons 989, 1011 and 1016 within domains II or IV were synonymous (codon nomenclature based on *Musca domestica VGSC* gene according to the accepted *kdr* codon nomenclature method). Genotyping of mosquitoes after deltamethrin bioassay was done in UBT and UYS populations due to the very small number of resistant individuals in the other four populations. At codon 1534, polymorphisms were detected in both populations with two mutated codons, F1534S and F1534 L. Both *kdr* mutations F1534S and F1534 L conferred protection against deltamethrin, with odds ratios of 33.6 and 9.3 for F1534S (*P* < 0.001), and odds ratios of 15.7 and 19.8 for F1534 L (*P* < 0.05) in the UBT and UYS populations, respectively (Table 3). In populations that underwent DDT resistant bioassay, mutated codons at the *VGSC* gene was detected in all populations except one rural population (RPL). However, *kdr* mutations were not significantly associated with mosquito resistance to DDT after a significance level adjustment for multiple comparisons (Table 4). No *kdr* mutation was detected in the laboratory susceptible populations.

**Table 3:** Association between mutations at codon 1534 of the voltage-gated sodium channel gene and phenotypic resistance to deltamethrin in two *Aedes albopictus* populations from Guangzhou, China

Area	Population	Phenotype	*n*	Genotype						Odds ratio (95% CI)		*P*-value of Fisher’s exact probability test	
				FF	FS	SS	FL	LL	SL	F1534S	F1534 L	F1534S	F1534 L
Urban	UBT	R	12	0	0	9	1	0	2	33.6 (4.26–263.97)	15.7 (1.46–168.08)	< 0.0001*	0.023*
		S	42	16	12	6	3	1	4				
	UYS	R	20	0	1	2	1	6	10	9.3 (1.94–44.55)	19.8 (4.17–93.99)	0.001*	< 0.0001*
		S	37	8	9	5	6	3	6				

*Abbreviations*: *R* resistant, *S* susceptible, *FF* homozygous phenylalanine/phenylalanine, *FS* heterozygotes phenylalanine/ leucine, *SS* homozygous serine/serine, *FL* heterozygotes phenylalanine/ leucine, *LL* homozygous leucine/ leucine, *SL* heterozygotes serine/leucine

^*^P < 0.05

**Table 4:** Genotyping results of the voltage-gated sodium channel gene at 1534 codon and association with resistance to DDT in five *Aedes albopictus* populations in Guangzhou, China. The significance threshold is *P* < 0.01 after Bonferroni correction for multiple testing

Area	Population	Phenotype	*n*	Genotype						Odds ratio (95% CI)		*P*–value of Fisher’s exact probability test	
				FF	FS	SS	FL	LL	LS	F1534S	F1534 L	F1534S	F1534 L
Urban	UBT	R	28	2	11	12	1	0	2	1.9 (0.94–3.96)	1.9 (0.38–9.34)	0.052	0.353
		S	46	15	9	18	1	0	3				
	UYS	R	39	5	9	7	6	1	11	2.3 (1.19–4.59)	2.2 (1.00–4.86)	0.010	0.040
		S	52	13	19	3	10	1	7				
Suburban	SBL	R	10	3	6	1	0	0	0	0.9 (0.31–2.51)	na	0.519	0.542
		S	29	11	9	7	1	0	1				
	SPX	R	10	0	1	8	0	0	1	4.2 (0.50–34.24)	2.0 (0.11–37.83)	0.143	0.589
		S	35	5	2	22	0	0	6				
Rural	RCD	R	13	8	3	2	0	0	0	1.0 (0.37–2.72)	na	0.587	0.346
		S	43	22	16	1	0	0	4				

*Abbreviations*: *F* wildtype F1534 allele, *S* F1534S allele, *L* F1534 L, *R* resistant, *S* susceptible, *na* not applicable, *FF* homozygous phenylalanine/phenylalanine, *FS* heterozygotes phenylalanine/ leucine, *SS* homozygous serine/serine, *FL* heterozygotes phenylalanine/ leucine, *LL* homozygous leucine/ leucine, *SL* heterozygotes serine/leucine

### Insecticide use and sales survey

Field surveys revealed that biological insecticides, pyrethroids, organophosphates and carbamates (except organochlorine) were used for agricultural and public health pest control in the study areas (Table 5). Overall, diverse types of insecticides are being used in the study sites. The pyrethroids-based insecticides were more commonly used than organophosphates. Pyrethrins (mainly cypermethrin and beta-cypermethrin) were the insecticides most frequently used in Guangzhou for community level adult *Ae. albopictus* control. However, the frequency of pyrethrin applications was higher in suburban and rural areas (5–7 times/week) than in urban areas (0–1 time/week). Pyrethrin and organophosphorus were the main insecticides used in suburban areas for agricultural purposes with a frequency of 1–2 times/week. In rural areas, organophosphorus and carbamates were the main insecticides used in agriculture with a frequency of 12 times/month.

**Table 5:** Survey of insecticide types and usage in three study settings in Guangzhou, China

	Mosquito status	*n*	Urban		Suburban		Rural	
			Insecticide	Frequency	Insecticide	Frequency	Insecticide	Frequency
Community usage	Adult	8	Pyrethrins: cypermethrin, beta-cypermethrin	1–2 times/month	Pyrethrin: cypermethrin; Organophosphates: DDVP	None or 1 time/year	Pyrethrin: cypermethrin	None or 1 time/year
	Larvae	8	Organophosphates: temephos, mevinphos, fenthion; Biological insecticides: bacillus sphaericus	1–2 times/month	–	–	–	–
Shop sold	Adult	10	Organophosphates: chlorpyrifos; carbamates: propoxur; Pyrethrin: prallethrin, cypermethrin, beta-cypermethrin, meperfluthrin, dimefluthrin, Es-Bioallethrin, tetramethrin	–	Pyrethrin: cypermethrin, beta-cypermethrin, permethrin, meperfluthrin, dimefluthrin, prallethrin; Carbamates: propoxur Organophosphates: chlorpyrifos	–	Organophosphates: DDVP, phoxim; Pyrethrin: meperfluthrin, dimefluthrin, Es-Bioallethrin, tetramethrin, cypermethrin, deyphenothrin	–
Resident usage	Adult	80	Pyrethrin: meperfluthrin, dimefluthrin, prallethrin, rich-d-transallethrin	None or 1 time/week(use at night)	Pyrethrin: meperfluthrin, dimefluthrin, prallethrin, rich-d-transallethrin, Es-Bioallethrin	5–7 times/week(use at night)	Pyrethrin: dimefluthrin, Es-Bioallethrin, rich-d-transallethrin, meperfluthrin, tetramethrin	5–7 times/week (use at night)
Agriculture usage	Rice field	10	–	–	–	–	Organophosphates: acephate	1–2 times/month
	Farm land	10	–	–	Pyrethrin: beta-cypermethrin, meperfluthrin; Organophosphates: DDVP, phoxim, Chlorpyrifos, dipterex	1–2 times/week	Organophosphates: DDVP, dipterex, acephate; Carbamates: methyl isocyanate	1 time/month

Organophosphorus (mainly temephos, mevinphos and fenthion) and biological insecticides (mainly *Bti*) were used for *Ae. albopictus* larval control but only in urban areas with a frequency of 1–2 times/month. No insecticide/larvicide was used for larval control in suburban or rural areas (Table 5).

## Discussion

Guangzhou is a dengue epidemic area and has been experiencing frequent outbreaks of dengue fever in China over the past 40 years. Also, it is prone to Zika virus outbreaks due to the presence of recently imported Zika cases in the area [46]. Thus, it is necessary to address the insecticide resistance problem of *Ae. albopictus* since insecticides were popularly used for mosquito control in the past 40 years. The present study is by far the most comprehensive research into insecticide resistance in *Ae. albopictus* mosquitoes from different ecological settings in Guangzhou. Two non-synonymous mutations at position 1534 of *kdr* gene domain III were identified with significant associations to deltamethrin resistance. Additionally, biochemical assays indicated that the three classes of detoxification enzymes may play a role in insecticide resistance in adult mosquitoes. Furthermore, insecticide usage surveys indicated a diverse use of insecticides in the study areas.

Our study area (Guangzhou) is in the subtropical region with a suitable climate for *Ae. albopictus* development and reproduction. Since the infamous and most deadly 2014 dengue epidemic in Guangdong Province, the city of Guangzhou has intensified vector control programs primarily through more frequent insecticide sprays [47]. In the present study, we illustrated that insecticide usage varied in different ecological settings (urban, suburban, and rural). Insecticide spray in urban areas was more frequent and intense than in suburban and rural areas. Adult *Ae. albopictus* populations from urban areas were more resistant to deltamethrin, DDT and propoxur than populations from rural areas, while no resistance to malathion was detected in the populations examined. Larval *Ae. albopictus* populations from urban areas were also more resistant to deltamethrin than populations from suburban and rural areas, whereas, all populations were susceptible to *Bti* and malathion.

According to the insecticide use survey, we observed frequent deltamethrin insecticide applications in the community, which coincided with elevated deltamethrin resistance. Usage of pyrethroids in urban areas was more frequent than in suburban and rural areas. DDT was used extensively in the 1960s in China for agricultural pest control, but was banned in the 1980s [15, 18]. Since the 1980s, especially in recent years, pyrethroids were massively used to control *Ae. albopictus* in China [29, 48]. The large scale mosquito control program within urban areas likely contributed to the increasing selection pressure on insecticide resistant *Ae. albopictus*. Wide usage of insecticide treated nets (ITNs) and long-lasting insecticidal nets (LLINs) in African countries has been linked to the rapid development of pyrethroids resistance in malaria vector mosquitoes over the past decade [49, 50]. The present study suggests that pyrethroid resistance is emerging in *Ae. albopictus* in our study area, and it is important to develop an appropriate insecticide resistance management plan. Meanwhile, there is an urgency to adopt alternative effective vector control methods that are not reliant on chemical insecticide such as odor-baited traps, larval resource reduction [20] and biological control [7], as well as new chemical insecticides [30].

Among all the populations from Guangzhou tested for *kdr* mutations, two mutations (F1534S and F1534 L) were detected, and these mutations were positively associated with pyrethroid resistance. This result is consistent with previous studies which found that F1534S mutation was correlated with the deltamethrin resistance [51–53]. Thus, monitoring the *kdr* mutation frequency may aid the surveillance of pyrethroid resistance in *Ae. albopictus*. In addition, we found significantly higher P450, GST and COE enzyme activities in the field mosquitoes. Literature has reported potential role of GST in DDT resistance [24, 54, 55] and P450s in pyrethroid resistance in mosquitoes, but the precise role of these detoxification enzymes in *Ae. albopictus* insecticide resistance needs further study.

An interesting finding from this study is the revealed patchy distribution of insecticide resistant *Ae. albopictus*. Within the urban and rural areas, mosquito populations differed considerably in resistance. One implication of this finding is that we need to monitor the insecticide resistance status in local mosquito populations, and develop efficient mosquito control strategies that take the patchy distribution of resistance into consideration. Currently, biological insecticides such as *Bti* are not frequently applied for mosquito control in China and no resistance has been detected, thus they can be considered as alternative insecticides for vector control.

## Conclusions

Our findings urgently call for timely surveillance of insecticide resistance as well as attention to the roles of metabolic detoxification enzymes and *kdr* mutations in insecticide resistant *Ae. albopictus*. The threat of dengue outbreak calls for an intensified and effective vector control program. Appropriate insecticide resistance management and additional vector control tools that are not reliant on synthetic insecticides are urgently needed to reduce dengue transmission.

## Additional file

Additional file 1: Table S1. Knockdown time (KDT) and mortality rate of *Aedes albopictus* populations from urban, suburban and rural settings in Guangzhou, China using the standard WHO tube susceptibility bioassay against four insecticides. (XLSX 12 kb)

## Abbreviations

*Bti*: *Bacillus thuringiensis israelensis*
COEs: Carboxylesterase
DDT: Dichloro-diphenyl-trichloroethane
GSTs: Glutathione S-transferase
kdr: Knockdown resistance
P450s: P450 monooxygenase
